# Supplementary material for: Mosaic chromosomal alterations in peripheral blood leukocytes of children in sub-Saharan Africa
Source: Nat Commun. 2023 Dec 6;14:8081. doi: 10.1038/s41467-023-43881-0 (PMC10700489; doi:10.1038/s41467-023-43881-0)
Supplement: Supplementary file 1 — Supplementary Information [file 41467_2023_43881_MOESM1_ESM.pdf]

Mosaic chromosomal alterations in peripheral blood leukocytes of children in sub-Saharan Africa

Weiyin Zhou *et al.*,

Contents

Tables 2

Table S1: Translocations detected in tumor WGS data using 4 SV callers for two BL cases with mCAs in matched peripheral blood samples *. 2

Table S2: Cell line fingerprinting in paired tumor-lymhoblastoid cell lines (LCLs) from corresponding normal (WW1-BL/LCLs and BL-2/IARC-304 LCL) to confirm their authenticity and genetic relationship 3

Table S3: Genes on chromosome X that are either mutated in BL or indirectly linked with BL via Epstein-Barr Virus (EBV) overlapped by mCAs and their association with BL case status 4

Table S4: List of oncogenes and significantly mutated genes on chromosome 1q in BL cases. 5

Table S5: Subjects with detected mCAs and estimated contamination ranges. 7

Table S6: The number and percentage of subjects that would be excluded based on different thresholds for sample call rates. 8

Figures 9

Figure S1. Type of autosomal mCAs (n=438) plotted by the proportion of abnormal cells (p) on the x-axis versus relative copy number estimated from log R ratio on the y-axis. 9

Results in 9

Figure S2. Autosomal mCAs detected in tumor but not in matched blood sample. 11

Figure S3. Autosomal mCAs detected in blood but not in matched tumor sample. 12

Figure S4. Circos plots of mosaic chromosomal alterations (mCAs) and their genomic location (GRCh37) 13

Figure S5. Frequency of mCAs by age groups in cancer-free African children from Uganda, Tanzania, and Kenya in the EMBLEM study, cancer-free adult men from Ghana, and cancer-free European ancestry individuals from the US Prostate, Lung, Colon, and Ovarian Cancer Study (PLCO). 14

Figure S6. Copy number changes in the BL cell line WW1 and three matching clones from several passages of the matching paired normal LCLs. 15

Figure S7. Quality control steps in mCAs analysis for quality control assessment. 16

Figure S8. Box plots displaying sample completion/call rates for all subjects in the analysis versus subjects with mCAs 17

Supplementary References 19

Tables

Table S1: Translocations detected in tumor WGS data using 4 SV callers for two BL cases with mCAs in matched peripheral blood samples *.

IDs	SV caller	chrom_a	start_a	end_a	chrom_b	start_b	end_b	supporting read pairs
**BLGSP-71-08-00197-01A**	svaba	chr8	127735359	127735359	chr14	105860000	105860000	16
**BLGSP-71-08-00197-01A**	svaba	chr8	127735385	127735385	chr14	105773965	105773965	25
**BLGSP-71-08-00200-01A**	svaba	chr8	127735047	127735047	chr14	105745097	105745097	9
**BLGSP-71-08-00200-01A**	svaba	chr8	127735080	127735080	chr14	105744421	105744421	18
**BLGSP-71-08-00197-01A**	manta	chr8	127735369	127735369	chr14	105860000	105860000	28
**BLGSP-71-08-00197-01A**	manta	chr8	127735377	127735377	chr14	105773961	105773961	38
**BLGSP-71-08-00200-01A**	manta	chr8	127735045	127735047	chr14	105745097	105745097	12
**BLGSP-71-08-00200-01A**	manta	chr8	127735080	127735082	chr14	105744419	105744419	23
**BLGSP-71-08-00197-01A**	delly	chr8	127735371	127735371	chr14	105860000	105860001	36
**BLGSP-71-08-00197-01A**	delly	chr8	127735377	127735377	chr14	105773963	105773964	28
**BLGSP-71-08-00200-01A**	delly	chr8	127735045	127735045	chr14	105745097	105745098	17
**BLGSP-71-08-00200-01A**	delly	chr8	127735080	127735080	chr14	105744419	105744420	8
**BLGSP-71-08-00197-01A**	breakdancer	chr8	127735305	127735305	chr14	105859999	105859999	28
**BLGSP-71-08-00197-01A**	breakdancer	chr8	127735523	127735523	chr14	105773963	105773963	21

Tumor-detected translocations were not identified in the corresponding peripheral blood DNA.

*Data from the Burkitt Lymphoma Genome Sequencing Project (BLGSP) that generated whole-genome sequencing (WGS) data for paired tumor-normal samples; genotype array data from normal samples were used in the current study of mCAs.

Table S2: Cell line fingerprinting in paired tumor-lymhoblastoid cell lines (LCLs) from corresponding normal (WW1-BL/LCLs and BL-2/IARC-304 LCL) to confirm their authenticity and genetic relationship.

The identity and relationship of the paired tumor-normal cultured cells was confirmed by short tandem repeat (STR) fingerprinting of 10

Cell line	Clone	Cell line name	AMEL	CSF1PO	D13S317	D16S539	D21S11	D5S818	D7S820	TH01	TPOX	vWA
Pair #1 (WW1-BL/LCL)												
WW1-BL		WW1-BL	X,Y	13.13	8.12	11.11*	30,30	9.11	11.11	8.8	11.11	16.18
WW1-LCL	Ulm2020	WW1-LCL-20	X,Y	13.13	8.12	12.12	30,32.2	9.11	11.11	8.8	11.11	16.18
WW1-LCL	Ulm2014	WW1-LCL-U14	X,Y	13.13	8.12	12.12	30,32.2	9.11	11.11	4.8	11.11	16.18
WW1-LCL	Berlin2006	WW1-LCL-06	X,Y	13.13	8.12	12.12	30,32.2	9.11	11.11	4.8	11.11	16.18
Pair #2 (BL2/IARC-304)												
BL-2			X,Y	11,13	11,12	12,12	29,31	12,12	8,12	6,6	8,11	15,16
IARC-304			X,Y	11,13	11,12	12,12	29,30	12,12	8,12	6,6	8,11	15,16

microsatellite markers (GenePrint 10 System, Promega). Samples labeled BL-2 and IARC-304 are from the same patient.

*The STR results showed uniparental disomy (UPD) for the marker D1S539 in the WW1 BL/LCLs pairs, which was corroborated by WGS data that revealed UPD on 16q in both the tumor and WW1-LCLs, i.e., the tumor sample and LCL lost the other parental allele other than the normal sample, indicating the origin of the tumor is independent of LCL clones.

Table S3: Genes on chromosome X that are either mutated in BL or indirectly linked with BL via Epstein-Barr Virus (EBV) overlapped by mCAs and their association with BL case status.

					**Female chrX mCAs**		**Deletion spanned gene**		**Fisher's Exact**		
**Gene_IDs**	Chromosome	Start	End	Length	BL cases counts (median CF)	Cancer-free controls (median CF)	BL cases counts (median CF)	Cancer-free controls counts (median CF)	OR	95%CI	P -value*
DDX3X	chrX	41192560	41223725	31166	13 (0.02)	29 (0.032)	11 (0.016)	16 (0.014)	4.32	0.74-47.05	0.09
KDM6A	chrX	44732420	44971857	239438	13 (0.02)	29 (0.032)	11 (0.016)	16 (0.014)	4.32	0.74-47.05	0.09
BTK	chrX	100604434	100645784	41351	13 (0.02)	29 (0.032)	12 (0.016)	15 (0.013)	10.66	1.27-510.82	0.015
XIAP	chrX	122993661	123047829	54169	13 (0.02)	29 (0.032)	12 (0.016)	15 (0.013)	10.66	1.27-510.82	0.015
SH2D1A	chrX	123480131	123507010	26880	13 (0.02)	29 (0.032)	12 (0.016)	15 (0.013)	10.66	1.27-510.82	0.015

*All statistical tests used were two-sided.

Table S4: List of oncogenes and significantly mutated genes on chromosome 1q in BL cases.

OncogeneName	GeneType	Chrom	Start	End	Length	# mCAs	median CF	Sample_IDs	Sample_IDs2	Sample_IDs3	Sample_IDs4	Sample_IDs5	Sample_IDs6	Sample_IDs7	Sample_IDs8	Sample_IDs9	Sample_IDs10	Sample_IDs11	Sample_IDs12	Sample_IDs13
NBPF12	protein-coding	chr1	146373856	146467639	93784	2	0.17625	S1	S2											
CHD1L	protein-coding	chr1	146644772	146767447	122676	3	0.1671	S1	S2	S3										
BCL9	protein-coding	chr1	147013270	147098020	84751	5	0.0304	S1	S2	S3	S4	S5								
PFN1P3	pseudo	chr1	148349256	148349657	401	5	0.0304	S1	S2	S3	S4	S5								
PLEKHO1	protein-coding	chr1	150121623	150132260	10638	7	0.0637	S1	S2	S3	S4	S5	S6	S7						
RPRD2*	protein-coding	chr1	150336586	150449041	112455	7	0.0637	S1	S2	S3	S4	S5	S6	S7						
MCL1	protein-coding	chr1	150547026	150552214	5189	7	0.0637	S1	S2	S3	S4	S5	S6	S7						
SETDB1	protein-coding	chr1	150898814	150937220	38407	7	0.0637	S1	S2	S3	S4	S5	S6	S7						
MLLT11	protein-coding	chr1	151032150	151040973	8824	7	0.0637	S1	S2	S3	S4	S5	S6	S7						
PSMB4	protein-coding	chr1	151372040	151374412	2373	7	0.0637	S1	S2	S3	S4	S5	S6	S7						
S100A8	protein-coding	chr1	153362507	153395059	32553	11	0.0304	S1	S2	S3	S4	S5	S6	S7	S8	S9	S10	S11		
S100A7	protein-coding	chr1	153430219	153433137	2919	11	0.0304	S1	S2	S3	S4	S5	S6	S7	S8	S9	S10	S11		
S100A4	protein-coding	chr1	153516094	153518282	2189	11	0.0304	S1	S2	S3	S4	S5	S6	S7	S8	S9	S10	S11		
ILF2	protein-coding	chr1	153634263	153643504	9242	11	0.0304	S1	S2	S3	S4	S5	S6	S7	S8	S9	S10	S11		
INTS3	protein-coding	chr1	153700542	153747284	46743	11	0.0304	S1	S2	S3	S4	S5	S6	S7	S8	S9	S10	S11		
HAX1	protein-coding	chr1	154245038	154248355	3318	11	0.0304	S1	S2	S3	S4	S5	S6	S7	S8	S9	S10	S11		
HAX1	protein-coding	chr1	154245038	154248355	3318	11	0.0304	S1	S2	S3	S4	S5	S6	S7	S8	S9	S10	S11		
ATP8B2	protein-coding	chr1	154298035	154323780	25746	11	0.0304	S1	S2	S3	S4	S5	S6	S7	S8	S9	S10	S11		
CKS1B	protein-coding	chr1	154947117	154951725	4609	12	0.02795	S1	S2	S3	S4	S5	S6	S7	S8	S9	S10	S11	S12	
MUC1	protein-coding	chr1	155158299	155162706	4408	12	0.02795	S1	S2	S3	S4	S5	S6	S7	S8	S9	S10	S11	S12	
MIR92B	ncRNA	chr1	155164967	155165063	97	12	0.02795	S1	S2	S3	S4	S5	S6	S7	S8	S9	S10	S11	S12	
FAM189B	protein-coding	chr1	155216995	155225274	8280	12	0.02795	S1	S2	S3	S4	S5	S6	S7	S8	S9	S10	S11	S12	
FDPS	protein-coding	chr1	155278538	155290457	11920	12	0.02795	S1	S2	S3	S4	S5	S6	S7	S8	S9	S10	S11	S12	
YY1AP1*	protein-coding	chr1	155629232	155658823	29592	12	0.02795	S1	S2	S3	S4	S5	S6	S7	S8	S9	S10	S11	S12	
RIT1	protein-coding	chr1	155867598	155881193	13596	12	0.02795	S1	S2	S3	S4	S5	S6	S7	S8	S9	S10	S11	S12	
SNORA80E	snoRNA	chr1	155889699	155889833	135	12	0.02795	S1	S2	S3	S4	S5	S6	S7	S8	S9	S10	S11	S12	
ARHGEF2	protein-coding	chr1	155916629	155949351	32723	12	0.02795	S1	S2	S3	S4	S5	S6	S7	S8	S9	S10	S11	S12	
NTRK1	protein-coding	chr1	156785541	156851642	66102	12	0.02795	S1	S2	S3	S4	S5	S6	S7	S8	S9	S10	S11	S12	
ETV3	protein-coding	chr1	157090982	157108383	17402	12	0.02795	S1	S2	S3	S4	S5	S6	S7	S8	S9	S10	S11	S12	
FCRL5/IRTA2	protein-coding	chr1	157483166	157522310	39145	12	0.02795	S1	S2	S3	S4	S5	S6	S7	S8	S9	S10	S11	S12	
FCRL4/IRTA1	protein-coding	chr1	157543538	157567870	24333	12	0.02795	S1	S2	S3	S4	S5	S6	S7	S8	S9	S10	S11	S12	
FCRL3/IRTA3	protein-coding	chr1	157646270	157670662	24393	13	0.0304	S1	S2	S3	S4	S5	S6	S7	S8	S9	S10	S11	S12	S13
FCRL2/IRTA4	protein-coding	chr1	157715522	157746922	31401	13	0.0304	S1	S2	S3	S4	S5	S6	S7	S8	S9	S10	S11	S12	S13
FCRL1/IRTA5	protein-coding	chr1	157764193	157789940	25748	13	0.0304	S1	S2	S3	S4	S5	S6	S7	S8	S9	S10	S11	S12	S13
NHLH1*	protein-coding	chr1	160336860	160342638	5778	13	0.0304	S1	S2	S3	S4	S5	S6	S7	S8	S9	S10	S11	S12	S13
NECTIN4	protein-coding	chr1	161040780	161059385	18606	13	0.0304	S1	S2	S3	S4	S5	S6	S7	S8	S9	S10	S11	S12	S13
DUSP12	protein-coding	chr1	161719557	161726954	7398	13	0.0304	S1	S2	S3	S4	S5	S6	S7	S8	S9	S10	S11	S12	S13
PBX1	protein-coding	chr1	164528596	164855284	326689	13	0.0304	S1	S2	S3	S4	S5	S6	S7	S8	S9	S10	S11	S12	S13
XCL1	protein-coding	chr1	168545710	168551315	5606	14	0.029175	S1	S2	S3	S4	S5	S6	S7	S8	S9	S10	S11	S12	S13
PRRC2C*	protein-coding	chr1	171454665	171562650	107985	14	0.0304	S1	S2	S3	S4	S5	S6	S7	S8	S9	S10	S11	S12	S13
ABL2	protein-coding	chr1	179068461	179198819	130359	13	0.0304	S1	S2	S3	S4	S5	S6	S7	S8	S9	S11	S12	S13	S14
RGL1*	protein-coding	chr1	183605181	183897685	292504	13	0.0355	S1	S2	S3	S4	S5	S6	S7	S8	S9	S10	S12	S13	S14
TPR	protein-coding	chr1	186280785	186344457	63673	12	0.0496	S1	S2	S3	S4	S5	S6	S7	S8	S9	S10	S13	S14	
MIR181B1	RNA gene	chr1	198828001	198828111	111	13	0.0345	S1	S2	S3	S4	S5	S6	S7	S8	S9	S13	S14	S15	S16
MIR181A1	RNA gene	chr1	198828172	198828282	111	13	0.0345	S1	S2	S3	S4	S5	S6	S7	S8	S9	S13	S14	S15	S16
KIF14	protein-coding	chr1	200520624	200589862	69239	13	0.0345	S1	S2	S3	S4	S5	S6	S7	S8	S9	S13	S14	S15	S16
KDM5B	protein-coding	chr1	202694312	202778598	84287	13	0.0345	S1	S2	S3	S4	S5	S6	S7	S8	S9	S13	S14	S15	S16
MDM4	protein-coding	chr1	204485506	204527248	41743	13	0.0345	S1	S2	S3	S4	S5	S6	S7	S8	S9	S13	S14	S15	S16
CNTN2	protein-coding	chr1	205012064	205047400	35337	13	0.0345	S1	S2	S3	S4	S5	S6	S7	S8	S9	S13	S14	S15	S16
NUAK2	protein-coding	chr1	205271190	205290919	19730	13	0.0345	S1	S2	S3	S4	S5	S6	S7	S8	S9	S13	S14	S15	S16
MIR135B	RNA gene	chr1	205417429	205417526	98	13	0.0345	S1	S2	S3	S4	S5	S6	S7	S8	S9	S13	S14	S15	S16
FAM72A	protein-coding	chr1	206137264	206155151	17888	13	0.0345	S1	S2	S3	S4	S5	S6	S7	S8	S9	S13	S14	S15	S16
IKBKE	protein-coding	chr1	206643585	206670223	26639	13	0.0345	S1	S2	S3	S4	S5	S6	S7	S8	S9	S13	S14	S15	S16
IL10	protein-coding	chr1	206940946	206945839	4894	13	0.0345	S1	S2	S3	S4	S5	S6	S7	S8	S9	S13	S14	S15	S16
INTS7	protein-coding	chr1	212113740	212209002	95263	11	0.0355	S1	S2	S3	S5	S6	S7	S8	S9	S13	S14	S16		
ATF3	protein-coding	chr1	212738675	212794119	55445	11	0.0355	S1	S2	S3	S5	S6	S7	S8	S9	S13	S14	S16		

* Genes implicated in BL tumor studies (see text)

The list of oncogenes is from the oncogene database (**https://ongene.bioinfo-minzhao.org/index.html**) and the significantly mutated BL genes are from Lopez et al [1-4].

Table S5: Subjects with detected mCAs and estimated contamination ranges.

mCAs	# subjects with mCAs	# mCA detected	call rate range	contamination range*
**Autosomal**	209	438	0.988 to 0.999	0.011 to 0.063
**Female chrX**	45	60	0.988 to 0.999	0.012 to 0.017
**Male chrY**	117	117	0.95 to 0.999	0.010 to 0.063

* From subjects with available contamination range estimates

Table S6: The number and percentage of subjects that would be excluded based on different thresholds for sample call rates.

	Participants					
Call Rate	Detected autosomal mCAs		Total autosomal mCAs	no mCAs		Total
	N	%		N	%	
< 97%	0	0	209	18	0.38	4753
< 98%	0	0	209	34	0.72	4753

	Detected female X mCAs		Total female X mCAs	no mCAs		Total females
	N	%		N	%	
< 97%	0	0	45	9	0.41	2185
< 98%	0	0	45	16	0.73	2185

	Detected male Y mCAs		Total male Y mCAs	no mCAs		Total males
	N	%		N	%	
< 97%	2	0.017	117	9	0.35	2568
< 98%	2	0.017	117	18	0.70	2568

Figures

Figure S1. Type of autosomal mCAs (n=438) plotted by the proportion of abnormal cells (p) on the x-axis versus relative copy number estimated from log R ratio on the y-axis.

Results in (**A**) are for 931 BL cases and in (**B**) 3822 controls in EMBLEM and Malawi; 'Source data are provided as a Source Data file.'.

A

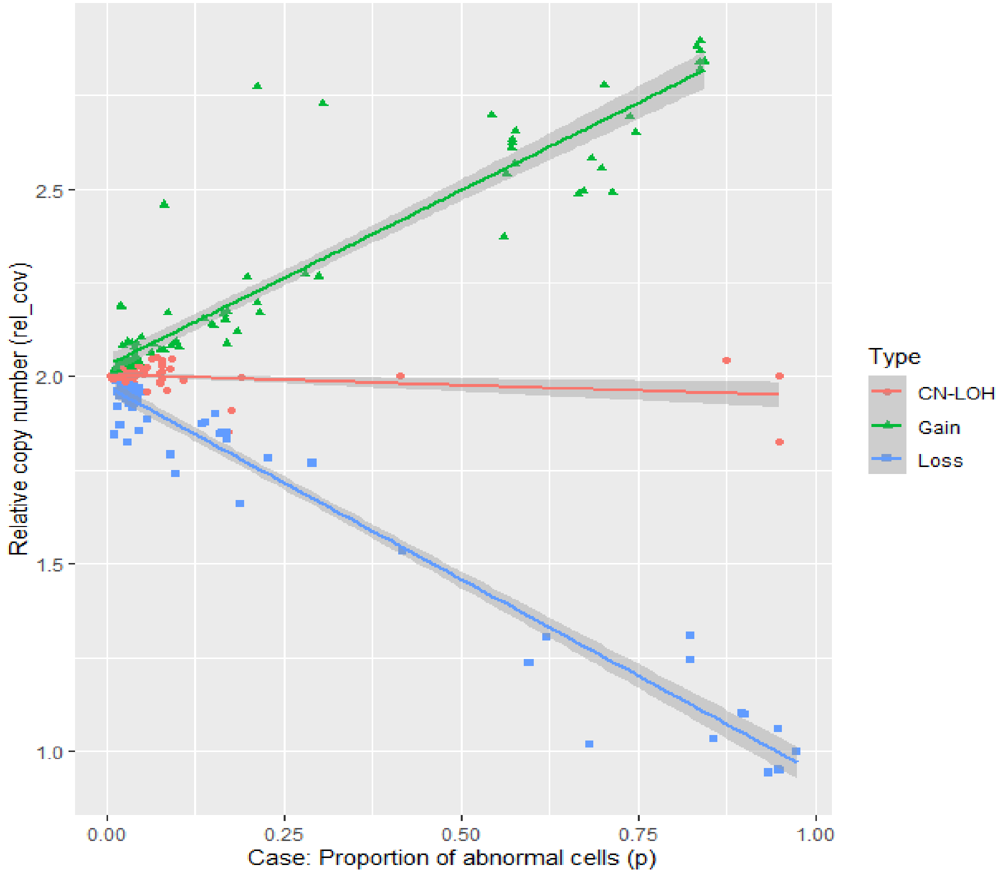

B

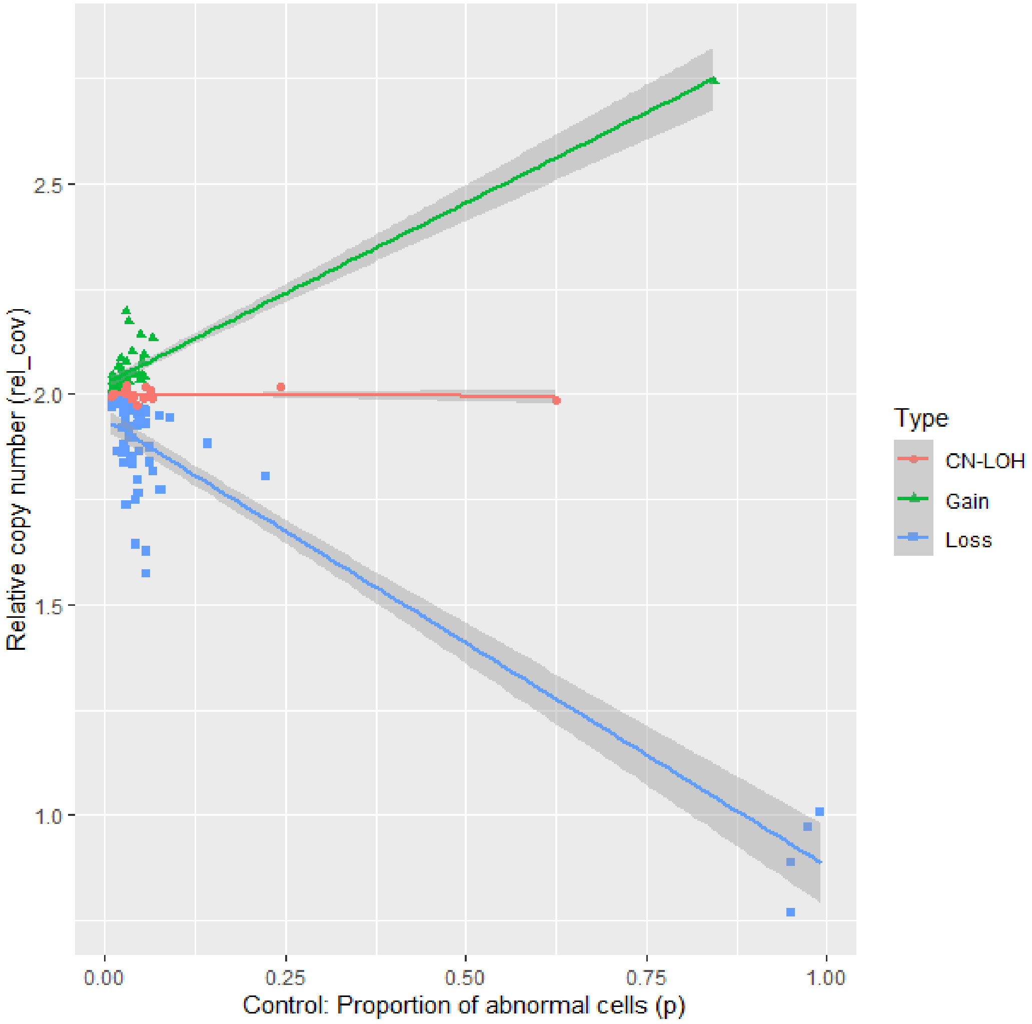

Relative copy number (rel_cov)
2.5
2.0
1.5
1.0
0.00
0.25
0.50
0.75
1.00
Control: Proportion of abnormal cells (p)
Type
CN-LOH
Gain
Loss

Figure S2. Autosomal mCAs detected in tumor but not in matched blood sample.

(**A**) Two chromosome 17 CNLOH events detected with cell fractions of 8.8% and 37% in WGS data of the same tumor. (**B**) These CNLOH events were not detected in SNP array data from peripheral blood leukocytes of the same subject. Upper panels: exponentiated Log R ratio (eLRR) of SNP data or Log depth of read coverage of WGS data; Bottom panels: phase B allele frequency (pBAF). Grey color indicates regions with a normal state, orange color indicates regions CNLOH with cell fraction (CF) = 8.8% while yellow indicates regions with CNLOH with CF = 37%. 'Source data are provided as a Source Data file.' .

A

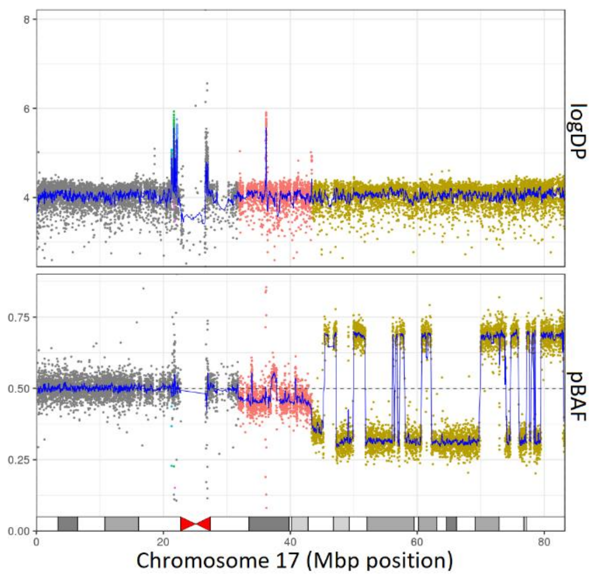

B

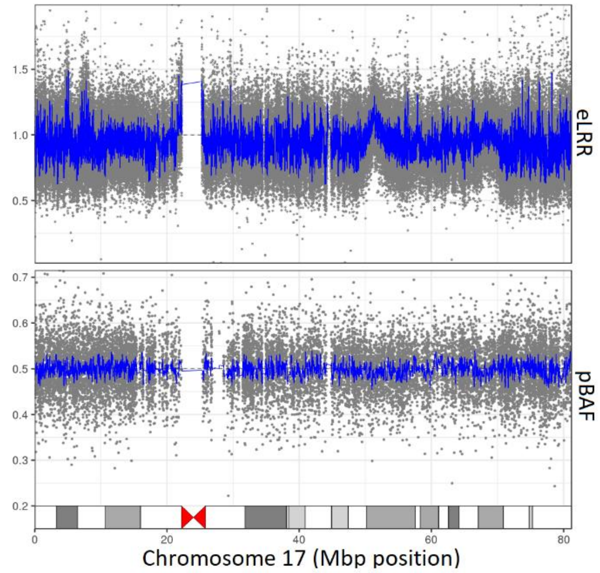

Figure S3. Autosomal mCAs detected in blood but not in matched tumor sample.

(**A**) One chromosome 21 CNLOH was detected in SNP array data from peripheral blood leukocytes with a cell fraction ~12%. (**B**) This CNLOH event was not detected in WGS tumor data of the same subject. Upper panels: exponentiated Log R ratio (eLRR) of SNP data or Log depth of read coverage of WGS data; Bottom panels: phase B allele frequency (pBAF), Grey color indicates regions with a normal state. Orange color indicates regions with CNLOH with cell fraction (CF) ~ 12%. 'Source data are provided as a Source Data file.' .

A **B**

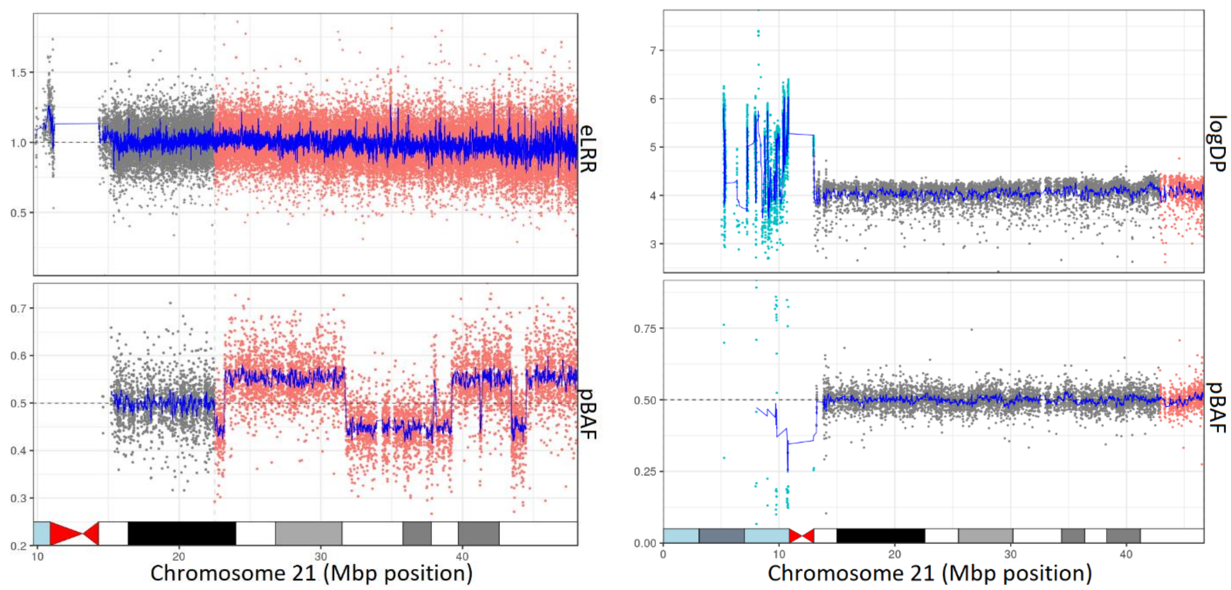

Figure S4. Circos plots of mosaic chromosomal alterations (mCAs) and their genomic location (GRCh37).

Outer rings are the autosomes 1 to 22. mCAs are shown in different colored areas: duplication events in green area; copy-neutral LOH events in blue area; copy-loss events in red area. (**A**) mCAs detected in blood cells of 701 BL cases in EMBLEM. (**B**) mCAs detected in blood cells of 674 healthy men in Ghana; 'Source data are provided as a Source Data file.'.

A **B**

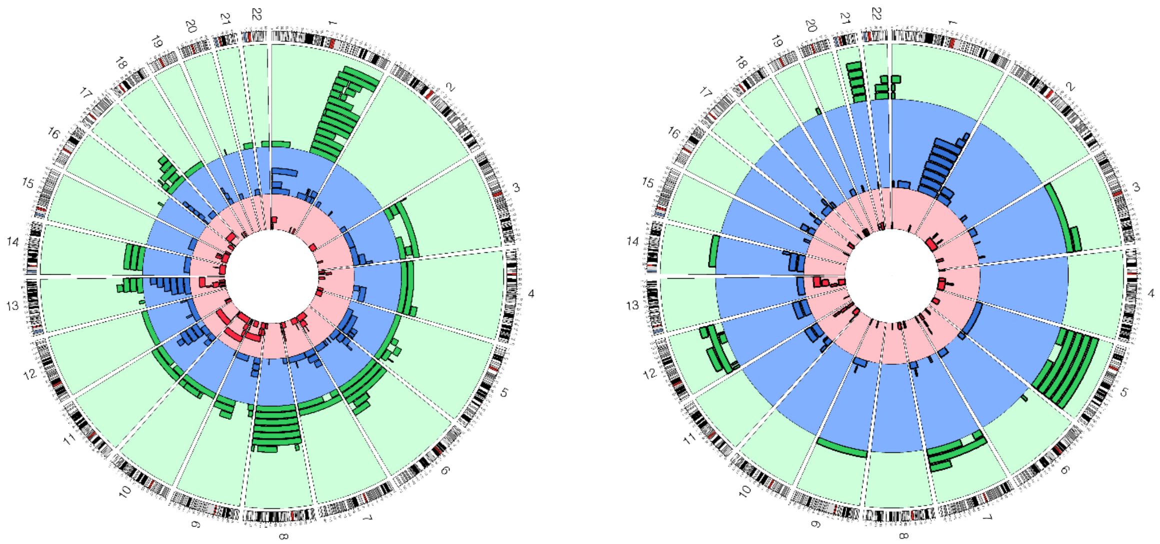

Figure S5. Frequency of mCAs by age groups in cancer-free African children from Uganda, Tanzania, and Kenya in the EMBLEM study, cancer-free adult men from Ghana, and cancer-free European ancestry individuals from the US Prostate, Lung, Colon, and Ovarian Cancer Study (PLCO).

Results are based on ~2.1 million genotyped, phased and analyzed using the same bioinformatic pipeline to detect mCAs in 3,645 cancer-free children in EMBLEM, 651 cancer-free adults in Ghana, and 2,618 cancer-free adults in PLCO. The frequency of mCAs is shown as bar charts, by 5-year age groups; the whiskers showing the 95% confidence interval of the estimate. The sample size for each age group is written below each age group bar; 'Source data are provided as a Source Data file.'.

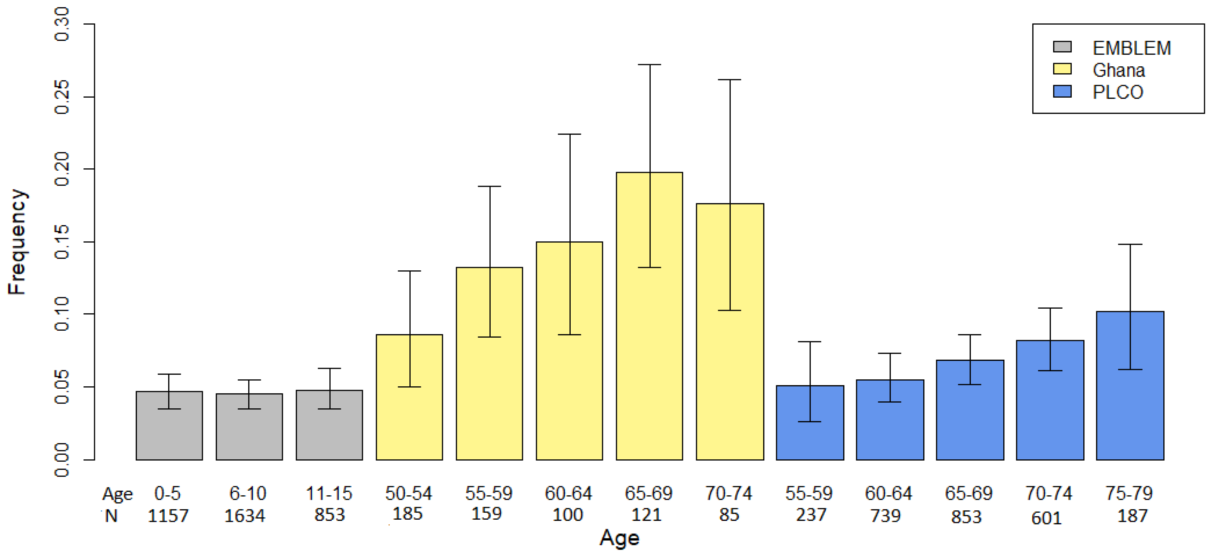

Figure S6. Copy number changes in the BL cell line WW1 and three matching clones from several passages of the matching paired normal LCLs.

Copy number profiles obtained from DNA methylation arrays. (A) Results for BL cell line WW1. (B) Results for different LCL clones created from the normal peripheral blood sample matching WW1 BL cell line: WW1-LCL-B06, WW1-LCL-U14, WW1-LCL-U20. For both A and B, the green colored dots in the upper panel represent gains, while red colored dots on the same panel represent losses of genetic material. The middle panel shows the results of the cytoScans. The colors (red, green, yellow, and blue) of the cytoscans represent different chromosomes and the WGS results are shown in the lower panel as Log2 in a whole-genome view, excluding sex chromosomes. (C) Results of a CytoScan Array showing chromosome 3q copy number changes in the WW1 BL and the three WW1 matching LCLs (WW1-LCL-B06, WW1-LCL-U14, WW1-LCL-U20). Colors represent different cell lines: violet-WW1-BL cell line; green-WW1-LCL-U20, orange-WW1-LCLU14, and blue-WW1-LCL-B06). Vertical dashed lines indicate the positions of the 3q gains, which vary in the genomic position in the WW1-BL tumor cell line versus the two WW1 LCLs (U20 and U14) in which they were detected; 'Source data are provided as a Source Data file.'.

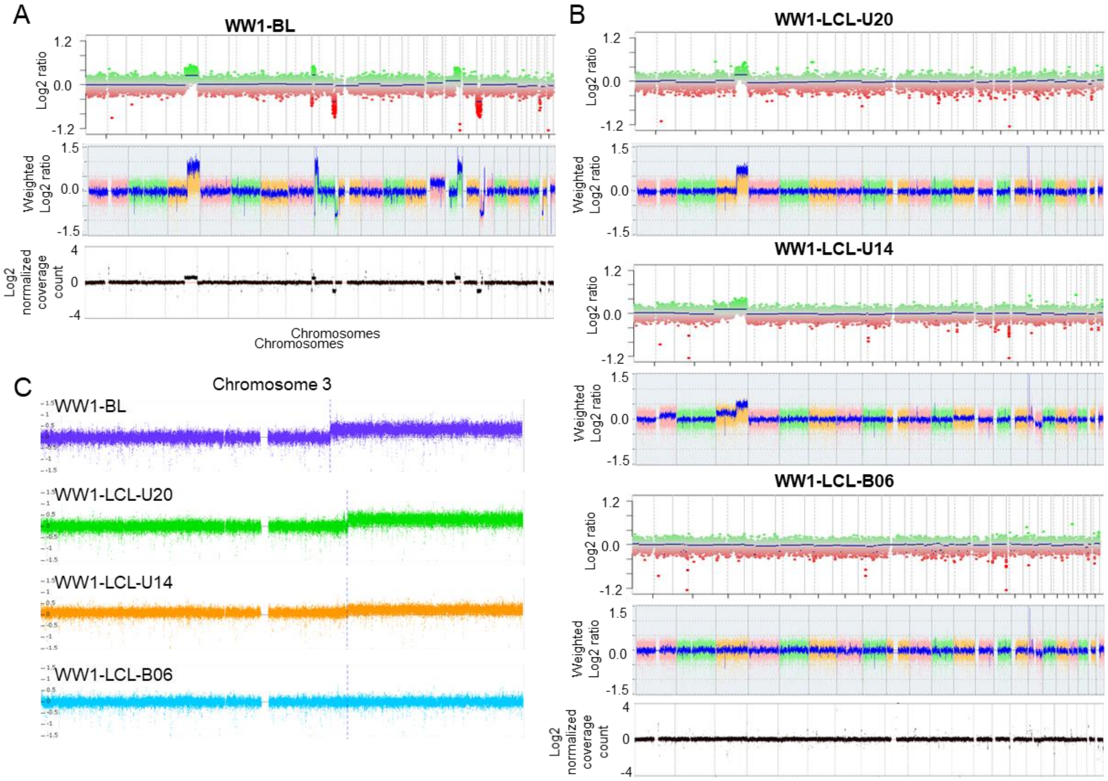

Figure S7. Quality control steps in mCAs analysis for quality control assessment. The flow chart shows a 10-step standard quality control pipeline applied to curate genotype data prior to analysis. Standard steps include filtering samples with low completion/call rates (<0.95), with high contamination rates (>=0.10), de-duplication, sex discordant samples, and loci with low (<0.95) completion rates prior to analysis.

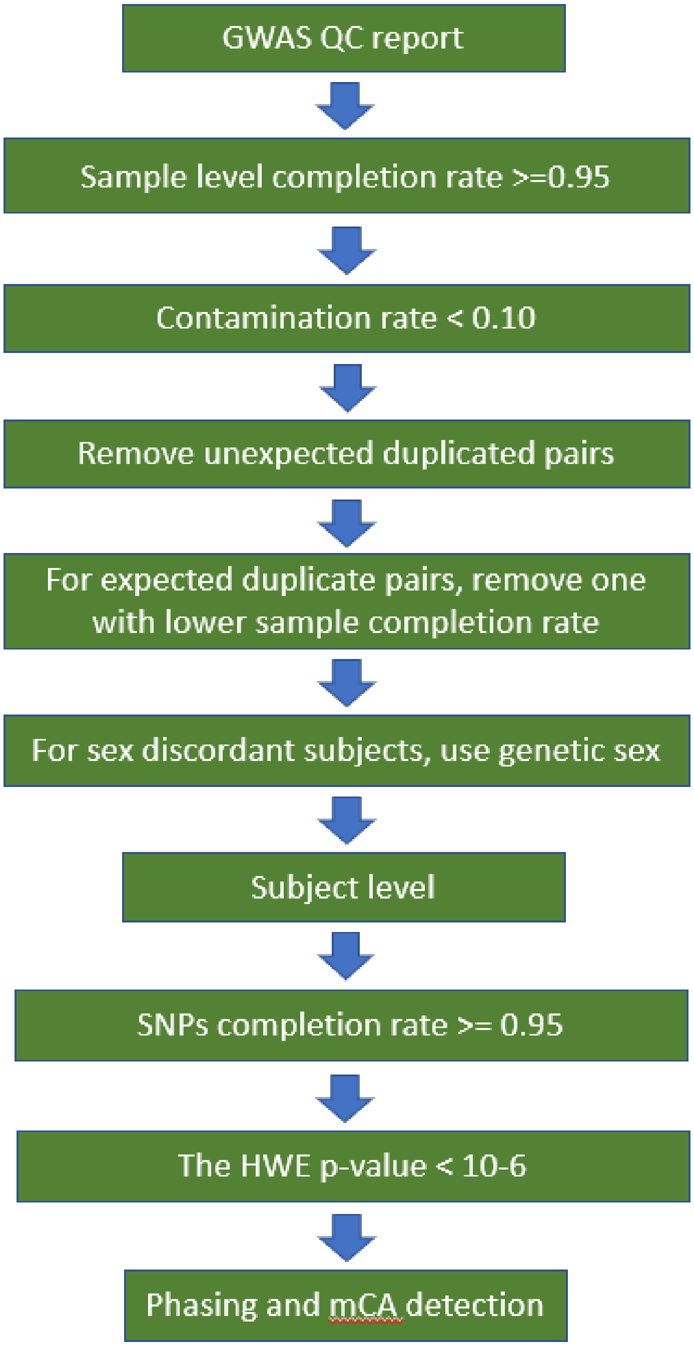

Figure S8. Box plots displaying sample completion/call rates for all subjects in the analysis versus subjects with mCAs

The box plots show the distributions of the sample completion/call rates all subjects (N=4,753) and for those with mCAs detected (n=209), stratified by sex (all females, N= 2,185 and females with mCAs n=45 and all males, N= 2,568, males with mCAs n=117). The two whiskers for each box plot mark the minima and maxima completion/call rates, and the dots beyond two whiskers are outlier completion/call rates. Analysis was limited to samples with a completion/call rate >=0.95. 'Source data are provided as a Source Data file.'

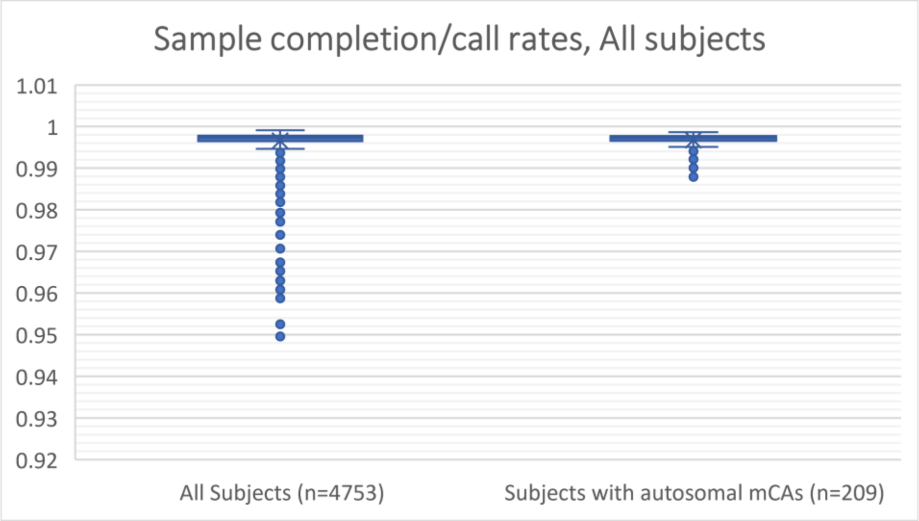

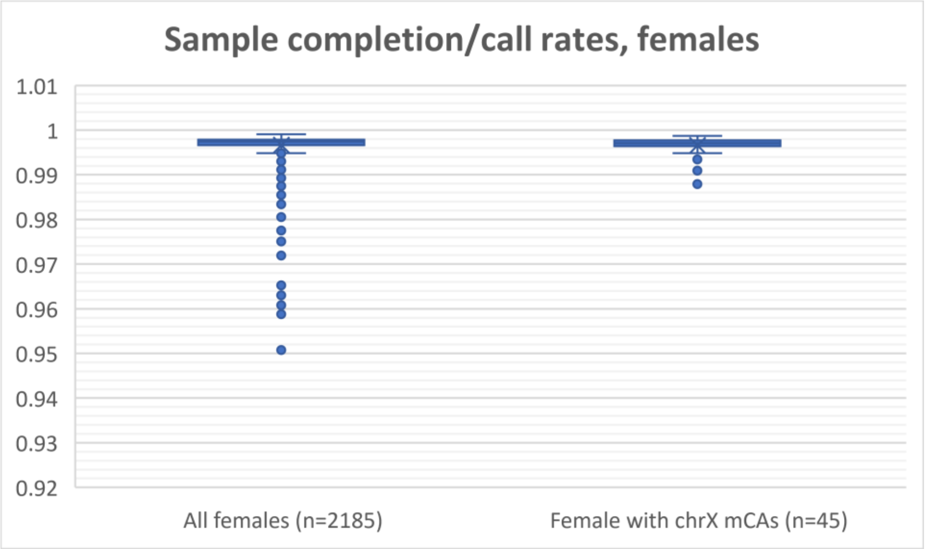

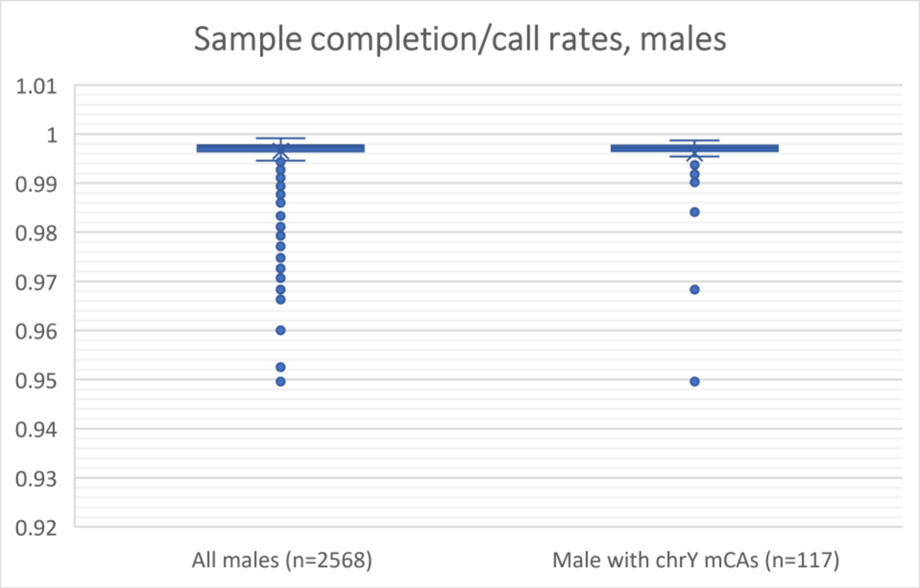
Sample completion/call rates, males
1.01
1
0.99
0.98
0.97
0.96
0.95
0.94
0.93
0.92
All males (n=2568)
Male with chrY mCAs (n=117)

Supplementary References

1. López, C. *et al.* Genomic and transcriptomic changes complement each other in the pathogenesis of sporadic Burkitt lymphoma. *Nat Commun* **10**, 1459 (2019).
2. Grande, B.M. *et al.* Genome-wide discovery of somatic coding and noncoding mutations in pediatric endemic and sporadic Burkitt lymphoma. *Blood* **133**, 1313-1324 (2019).
3. Abate, F. *et al.* Distinct Viral and Mutational Spectrum of Endemic Burkitt Lymphoma. *PLoS Pathog* **11**, e1005158 (2015).
4. Thomas, N. *et al.* Genetic subgroups inform on pathobiology in adult and pediatric Burkitt lymphoma. *Blood* **141**, 904-916 (2023).
