# Supplementary material for: Mosaic chromosomal alterations in peripheral blood leukocytes of children in sub-Saharan Africa
Source: Nat Commun. 2023 Dec 6;14:8081. doi: 10.1038/s41467-023-43881-0 (PMC10700489; doi:10.1038/s41467-023-43881-0)
Supplement: Supplementary file 3 — Reporting Summary [file 41467_2023_43881_MOESM3_ESM.pdf]

nature portfolio

Corresponding author(s): SM Mbulaiteye

Last updated by author(s): Nov 17, 2023

Reporting Summary

Nature Portfolio wishes to improve the reproducibility of the work that we publish. This form provides structure for consistency and transparency in reporting. For further information on Nature Portfolio policies, see our Editorial Policies and the Editorial Policy Checklist.

Statistics

For all statistical analyses, confirm that the following items are present in the figure legend, table legend, main text, or Methods section.

n/a	Confirmed	
☐	☒	The exact sample size (*n*) for each experimental group/condition, given as a discrete number and unit of measurement
☐	☒	A statement on whether measurements were taken from distinct samples or whether the same sample was measured repeatedly
☐	☒	The statistical test(s) used AND whether they are one- or two-sided<br>*Only common tests should be described solely by name; describe more complex techniques in the Methods section.*
☐	☒	A description of all covariates tested
☐	☒	A description of any assumptions or corrections, such as tests of normality and adjustment for multiple comparisons
☐	☒	A full description of the statistical parameters including central tendency (e.g. means) or other basic estimates (e.g. regression coefficient) AND variation (e.g. standard deviation) or associated estimates of uncertainty (e.g. confidence intervals)
☐	☒	For null hypothesis testing, the test statistic (e.g. *F*, *t*, *r*) with confidence intervals, effect sizes, degrees of freedom and *P* value noted<br>*Give P values as exact values whenever suitable.*
☒	☐	For Bayesian analysis, information on the choice of priors and Markov chain Monte Carlo settings
☒	☐	For hierarchical and complex designs, identification of the appropriate level for tests and full reporting of outcomes
☒	☐	Estimates of effect sizes (e.g. Cohen's *d*, Pearson's *r*), indicating how they were calculated

Our web collection on statistics for biologists contains articles on many of the points above.

Software and code

Policy information about availability of computer code

Data collection	Questionnaire data for EMBLEM was processed using DataFax under the NIAID Office of Cyber Infrastructure and Computational Biology clinical research support program. No other software was used for data collection.
Data analysis	1. MoChA software (v2021-05-14) (https://github.com/freeseek/mocha) was used to detect mCAs in the SNP array and WGS data<br>2. Eagle2 software for phasing to infer haplotypes for SNP array data and SHAPEIT4 software for phasing to infer haplotypes in WGS data<br>3. MoCCA-SV structural variation detection pipeline (https://github.com/NCI-CGR/MoCCA-SV) with four callers (svaba, breakdancer, manta, delly) was used to confirm presence of IG::MYC translocations in the WGS tumor data and to search for presence of tumor translocations in the WGS from the corresponding normal sample.<br>4. Generalized linear models to calculate odds ratios (OR) with 95% confidence intervals (CI), Mantel-Haenszel stratified analysis to assess homogeneity of associations of BL with mCAs across the countries; Fisher's exact test was used when cell numbers were small, and two-sample t-tests.<br>5. Stringtie used for calculation of strand-specific TPMs.

For manuscripts utilizing custom algorithms or software that are central to the research but not yet described in published literature, software must be made available to editors and reviewers. We strongly encourage code deposition in a community repository (e.g. GitHub). See the Nature Portfolio guidelines for submitting code & software for further information.

Data

Policy information about availability of data

All manuscripts must include a data availability statement. This statement should provide the following information, where applicable:

- Accession codes, unique identifiers, or web links for publicly available datasets
- A description of any restrictions on data availability
- For clinical datasets or third party data, please ensure that the statement adheres to our policy

The previously published data from the Burkitt Lymphoma Genome Sequencing Project are available under restricted access in the Genomic Data Commons under accession link [phs000235.v20.p6] for sub-study [phs000527.v18.p6]. The previously published data from the Ghana Prostate Healthy Study data are available under restricted access in dbGaP [phs000838.v1.p1]. The genetic data generated in this study are genotypes of participants in the EMBLEM and Malawi studies measured using Illumina Infinium Omni5Exome BeadChip arrays are novel. The data are under controlled access (requires IRB approval and are limited to not-for-profit research). Readers can access the data by applying via dbGaP under accession link [phs001705.v1.p1]. The remaining data reported in the article are available within the Article, Supplementary Information, or Source Data file provided with this paper.

Human research participants

Policy information about studies involving human research participants and Sex and Gender in Research.

Reporting on sex and gender	Results are reported by sex to assess the biologic effects of sex on mCA. Sex was collected on questionnaire and then confirmed by genetic testing based on the identifiler. When reported sex was discordant with genetic sex (in 31 instances), the genetic sex was considered the sex of that individual. We made decision after reviewing multiple forms where sex was recorded, usually by different observers who recorded the sex from the guardians, and noting that sex reported on these forms was usually consistent. Karyotypes of some of the discordant individuals revealed abnormalities such as XO, XXY, which suggested to us that some children had ambiguous sex , but parents in villages usually assign sex and use consistently.
Population characteristics	The study includes 4, 753 children (931 with Burkitt lymphoma) under 16 years enrolled in Uganda, Tanzania, Kenya, and Malawi. 2,568 (54%) of the children were male, 2,185 (46%) were female. The mean age was 7.43 years (standard deviation (3.59), with 1,576 (33%) aged 0-5 years, 2099 (44%) 6-10 years, 1064 (22%)11-15 years. Age was not recorded in 14 children. Asymptomatic Plasmodium falciparum parasites were detected 2,155 (45%) of the children and negative in 2,527 (53%) of the children, and data were missing for 71 (1%).<br><br>Sensitivity analyses performed on a combined dataset from EMBLEM, Ghana, and Prostate, Lung, Colorectal, Ovarian Cancer Screening Trial (PLCO) studies with 14,053 subjects.
Recruitment	The participants are from two studies: a) The EMBLEM study was a population-based case-control study that enrolled participants from six regions, two neighboring in Uganda along the River Nile in northern Uganda and four neighboring in Tanzania and Kenya on the southern shores of Lake Victoria during 2010-2016. The BL cases were diagnosed at participating hospitals, and diagnosed by histology or cytology (61% of cases) and compatible clinical and laboratory investigation in the remainder. Controls were apparently healthy children enrolled from 300 villages randomly selected in the study region. The controls were enrolled either in their village as matched controls (selected based on age and sex distribution of BL cases) or as survey controls, where all children in the selected village were enrolled, or as healthy facility controls for children attending village health facilities for minor ailments. The main biases for cases was incomplete ascertainment of cases, while for controls, the main concern was representativeness of the controls for children in the study area.<br>b) The Malawi study was hospital-based conducted at a tertiary-level hospital where all children were attending because of suspected cancer during 2005-2008. This is a case-case study where children with BL were compared to children with other cancers. BL diagnosis was based on local histology or cytology, or compatible clinical and laboratory investigation. Children with other solid tumors were used as controls. Those with Kaposi sarcoma, HIV positivity, and lymphoid or leukemic diagnoses were excluded from the controls.<br>c) The Ghana samples were from the Ghana Prostate Health Study. Participants were a random sample of 50-74 year old men enrolled from Greater Accra in Ghana between 2004-2006.
Ethics oversight	Ethical approval for EMBLEM was granted by Uganda Virus Research Institute (GC/127), Uganda National Council for Science and Technology (HS-816), Tanzania National Institute for Medical Research (NIMR/HQ/R.8c/Vol. IX/1023), Moi University/Moi Teaching and Referral Hospital (000536), and National Cancer Institute (10-C-N133) ethics committees. Ethical approval for the original Malawi Infections and Childhood Cancer study was granted by the Malawi College of Medicine (P.03/04/277R) and Oxford University. Because the original Malawi Infections and Childhood Cancer study did not request participants to consent to genetic testing, special ethical approval to conduct genetic testing was obtained from the National Health Sciences Research Committee (2405). The Noguchi Memorial Institute for Medical Research Institutional Review Board (001/01-02) and the NCI (02CN240) approved the Ghana Prostate Health Survey. Written informed consent was obtained from guardians of all child participants in EMBLEM and Malawi studies, and from adult participants in the Ghana prostate Health study. Written marked assent was obtained from children >7 years old in the EMBLEM study only. The NCI special studies ethics review board gave ethical approval to conduct the Prostate, Lung, Colorectal, Ovarian Cancer Screening Trial (PLCO) study.

Note that full information on the approval of the study protocol must also be provided in the manuscript.

Field-specific reporting

Please select the one below that is the best fit for your research. If you are not sure, read the appropriate sections before making your selection.

☒ Life sciences ☐ Behavioural & social sciences ☐ Ecological, evolutionary & environmental sciences

For a reference copy of the document with all sections, see nature.com/documents/nr-reporting-summary-flat.pdf

Life sciences study design

All studies must disclose on these points even when the disclosure is negative.

Sample size	The current study was conducted in a dataset prepared for GWAS of BL. The BL GWAS was designed to have 80% power to detect minimum odds ratios ranging from 0.39 to 0.57 assuming a baseline prevalence in cases of 5% to 25% and 12% to 37% in the controls. No power calculations were done for mCA studies because the frequency of mCAs in African populations is not known. Not calculating power is not unusual for such studies. The current was descriptive to investigate the frequency of mCAs in African children, and the regions on chromosomes where mCAs occur and provides baseline results that can be used to calculate power of future studies.
Data exclusions	We excluded 746 samples (611 subjects were excluded because of lack of genotype array data, and 135 subjects because of BL diagnosis was not confirmed, were HIV positive, or had Kaposi sarcoma) yielding an analytical dataset of 4,753.
Replication	We performed a sensitivity analysis was on 7,873 cancer-free individuals in EMBLEM (3,645), Ghanaian men (651), and European-ancestry adults from the Prostate, Lung, Colorectal and Ovarian Cancer Screening Trial (PLCO) (2618 cancer-free controls, 95.4% males) with ~2.1 million shared genotyped markers using the same Illumina Infinium chemistry and analyzed in the same facility using the same pipelines to confirm patterns observed in African subjects and compare results with individuals in the US.<br><br>For our tumor studies, we performed analysis in 13 patients in EMBLEM with whole genome sequencing (WGS) data from paired samples of peripheral blood and tumor, as well as genotype array data from the same peripheral blood sample. This subset allowed us to evaluate to what extent mCAs detected in peripheral blood might be due to tumor cells leaking into circulation. We confirmed that IG::MYC translocations detected in tumor cells were not detected in peripheral blood, including in two patients in whom mCAs were detected in normal samples using genotype arrays. We accessed two paired BL tumor-derived and corresponding non-tumor-derived lymphoblastoid cell lines (LCLs) to supplement our primary tumor-normal paired samples. The results of similar abnormalities in both tumor and normal samples supported our interpretation that mCAs being detected in early tumor clones, and not being sorely due to tumor contamination.
Randomization	The study is descriptive, thus the randomization was not done.
Blinding	Genetic testing was done with lab staff blinded about the case-control status of the samples, as well as the other characteristics of the samples.

Reporting for specific materials, systems and methods

We require information from authors about some types of materials, experimental systems and methods used in many studies. Here, indicate whether each material, system or method listed is relevant to your study. If you are not sure if a list item applies to your research, read the appropriate section before selecting a response.

Materials & experimental systems

n/a	Involved in the study	
☒	☐	Antibodies
☒	☐	Eukaryotic cell lines
☒	☐	Palaeontology and archaeology
☒	☐	Animals and other organisms
☐	☒	Clinical data
☒	☐	Dual use research of concern

Methods

n/a	Involved in the study	
☒	☐	ChIP-seq
☒	☐	Flow cytometry
☒	☐	MRI-based neuroimaging

Clinical data

Policy information about clinical studies

All manuscripts should comply with the ICMJE guidelines for publication of clinical research and a completed CONSORT checklist must be included with all submissions.

Clinical trial registration	NCT01196520
Study protocol	The EMBLEM study protocol can be accessed at: https://emblem.cancer.gov/resources/index.html. The BLGSP protocol can be accessed at: https://ocg.cancer.gov/programs/cgci/projects/burkitt-lymphoma.

March 2021

Data collection	Data collection in EMBLEM was conducted in six regions (2 neighboring in northern Uganda on opposite sides of the R. Nile and four neighboring in Tanzania and Kenya on the southern shores of L. Victoria) during 2010-2016. BL cases were enrolled at participating hospitals, while the controls were enrolled from 300 villages randomly selected from the six regions. The Malawi cases were enrolled at the Queen Elizabeth Hospital in Blantyre during 2005-2008. All children were referred for suspected cancer. BL cases were defined based on histological or cytological diagnosis with clinical and supportive laboratory results. Venous blood was collected before cancer treatment.
Outcomes	The mCAs were detected using MoChA software (v2021-05-14) (https://github.com/freeseek/mocha) in the SNP array and WGS data . The participants were grouped as BL cases based on local histology, cytology, or clinical and laboratory diagnosis. All other participants were used as controls. In Malawi, the controls were comprised of children with other cancers.
